# Trainer in a pocket - proof-of-concept of mobile, real-time, foot kinematics feedback for gait pattern normalization in individuals after stroke, incomplete spinal cord injury and elderly patients

**DOI:** 10.1186/s12984-018-0389-4

**Published:** 2018-05-29

**Authors:** Daniel Schließmann, Maria Nisser, Christian Schuld, Till Gladow, Steffen Derlien, Laura Heutehaus, Norbert Weidner, Ulrich Smolenski, Rüdiger Rupp

**Affiliations:** 1 Spinal Cord Injury Center, Heidelberg University Hospital, 69118 Heidelberg, Germany; 2 Institute for Physiotherapy, University Hospital Jena, 07747 Jena, Germany

**Keywords:** Feedback, Gait, Rehabilitation, Therapy, Incomplete spinal cord injury, Stroke, Elderly, Inertial measurement units, Wearable sensor

## Abstract

**Background:**

Walking disabilities negatively affect inclusion in society and quality of life and increase the risk for secondary complications. It has been shown that external feedback applied by therapists and/or robotic training devices enables individuals with gait abnormalities to consciously normalize their gait pattern. However, little is known about the effects of a technically-assisted over ground feedback therapy. The aim of this study was to assess whether automatic real-time feedback provided by a shoe-mounted inertial-sensor-based gait therapy system is feasible in individuals with gait impairments after incomplete spinal cord injury (iSCI), stroke and in the elderly.

**Methods:**

In a non-controlled proof-of-concept study, feedback by tablet computer-generated verbalized instructions was given to individuals with iSCI, stroke and old age for normalization of an individually selected gait parameter (stride length, stance or swing duration, or foot-to-ground angle). The training phase consisted of 3 consecutive visits. Four weeks post training a follow-up visit was performed. Visits started with an initial gait analysis (iGA) without feedback, followed by 5 feedback training sessions of 2–3 min and a gait analysis at the end. A universal evaluation and FB scheme based on equidistant levels of deviations from the mean normal value (1 level = 1 standard deviation (SD) of the physiological reference for the feedback parameter) was used for assessment of gait quality as well as for automated adaptation of training difficulty. Overall changes in level over iGAs were detected using a Friedman’s Test. Post-hoc testing was achieved with paired Wilcoxon Tests. The users’ satisfaction was assessed by a customized questionnaire.

**Results:**

Fifteen individuals with iSCI, 11 after stroke and 15 elderly completed the training. The average level at iGA significantly decreased over the visits in all groups (Friedman’s test, *p* < 0.0001), with the biggest decrease between the first and second training visit (4.78 ± 2.84 to 3.02 ± 2.43, *p* < 0.0001, paired Wilcoxon test). Overall, users rated the system’s usability and its therapeutic effect as positive.

**Conclusions:**

Mobile, real-time, verbalized feedback is feasible and results in a normalization of the feedback gait parameter. The results form a first basis for using real-time feedback in task-specific motor rehabilitation programs.

**Trial registration:**

DRKS00011853, retrospectively registered on 2017/03/23.

**Electronic supplementary material:**

The online version of this article (10.1186/s12984-018-0389-4) contains supplementary material, which is available to authorized users.

## Background

Walking disabilities negatively affect inclusion in society and quality of life [1–3]. Furthermore, a non-physiological gait pattern increases the risk of joint overuse resulting in pain [4] and - in the long run - osteoarthritis [5]. These secondary complications contribute additionally to the vicious cycle of walking disabilities and reduction in physical activity, which results in an increased risk for cardiovascular diseases [6]. Walking restrictions are present in patients with injuries of the central nervous system, such as stroke [7] or incomplete spinal cord injury (iSCI) [8, 9] and the associated sensorimotor and specifically proprioceptive impairments, or in aged individuals [10]. Several factors of rather diffuse origin contribute to walking disabilities in the elderly, among them various neurological and orthopedic disease conditions [11]. Similar to stroke and iSCI, sensory impairments have a negative impact on balance and walking ability [12, 13]. As afferent feedback is important for motor control in a variable environment [14] as well as for motor learning [15], extrinsic feedback (feedback from an external source, referred to as “feedback” in the following) is implemented in current gait rehabilitation regimes [16]. Feedback provides an individual with the ability to consciously close the sensorimotor control loop and motivates to focus on the task thereby increasing compliance [17].

In a feasibility study using a treadmill-based, real-time, optical motion analysis system, individuals with iSCI and reduced knee flexion during swing phase (“stiff-knee gait” like walking pattern) were able to normalize their gait pattern when abstract visual feedback of a weighted distance to the physiological reference was presented [18]. Surprisingly, the study participants maintained this effect even without feedback after the therapy [19]. While little is known about the long-term effect of feedback therapies in patients with neurological gait disorders, it has been shown in patients without neurological impairments, e.g. with patellofemoral pain syndrome, that function and medical complications such as pain improve with feedback-initiated normalization of the knee joint kinematics [20].

Optical, marker-based motion capture systems in combination with a treadmill are considered the gold standard for instrumented gait analysis and constitute an excellent platform for studies involving feedback, as they provide accurate [21], objective movement data [22], and a standardized setup concerning e.g. walking speed or number of steps. However, preparation times are high and gait kinetics on a treadmill are different from walking on even ground [23]. Inertial measurement unit (IMU)-based sensor systems represent inexpensive options compared to marker-based gait analysis systems and provide an easy-to-set-up possibility for feedback training in a natural environment [24–26]. Wearable sensors are commonly used for offline sensing / tracking [27, 28]. Some studies applied these sensors for balance training [29–31] or gait symmetry training [32]. Only a few mobile systems provide real-time feedback for use in a therapeutic setting [33]. Most of these systems are designed for providing feedback of a single gait parameter specific to a certain patient group such as Parkinson’s disease [34] or knee osteoarthritis [31, 35, 36]. Recently, shoe-mounted inertial sensors were applied to estimate and increase gait quality in aged adults by measuring angular velocity and giving binary real-time feedback [37]. So far, to our knowledge no study has provided proof-of-concept of an IMU-based real-time feedback training on the normalization of selected gait parameters during overground walking in persons with gait disorders of various etiologies.

Therefore, the aim of this pre-post-intervention, proof-of-concept, prospective cohort study was to assess whether automatic real-time verbalized feedback provided by a shoe-mounted IMU-based gait therapy system is feasible in individuals with gait impairments after iSCI, stroke and in the elderly. We hypothesized that with this feedback system the intentional normalization of an individually selected gait parameter is possible.

## Material and methods

### Feedback implementation

**Fig. 1:**
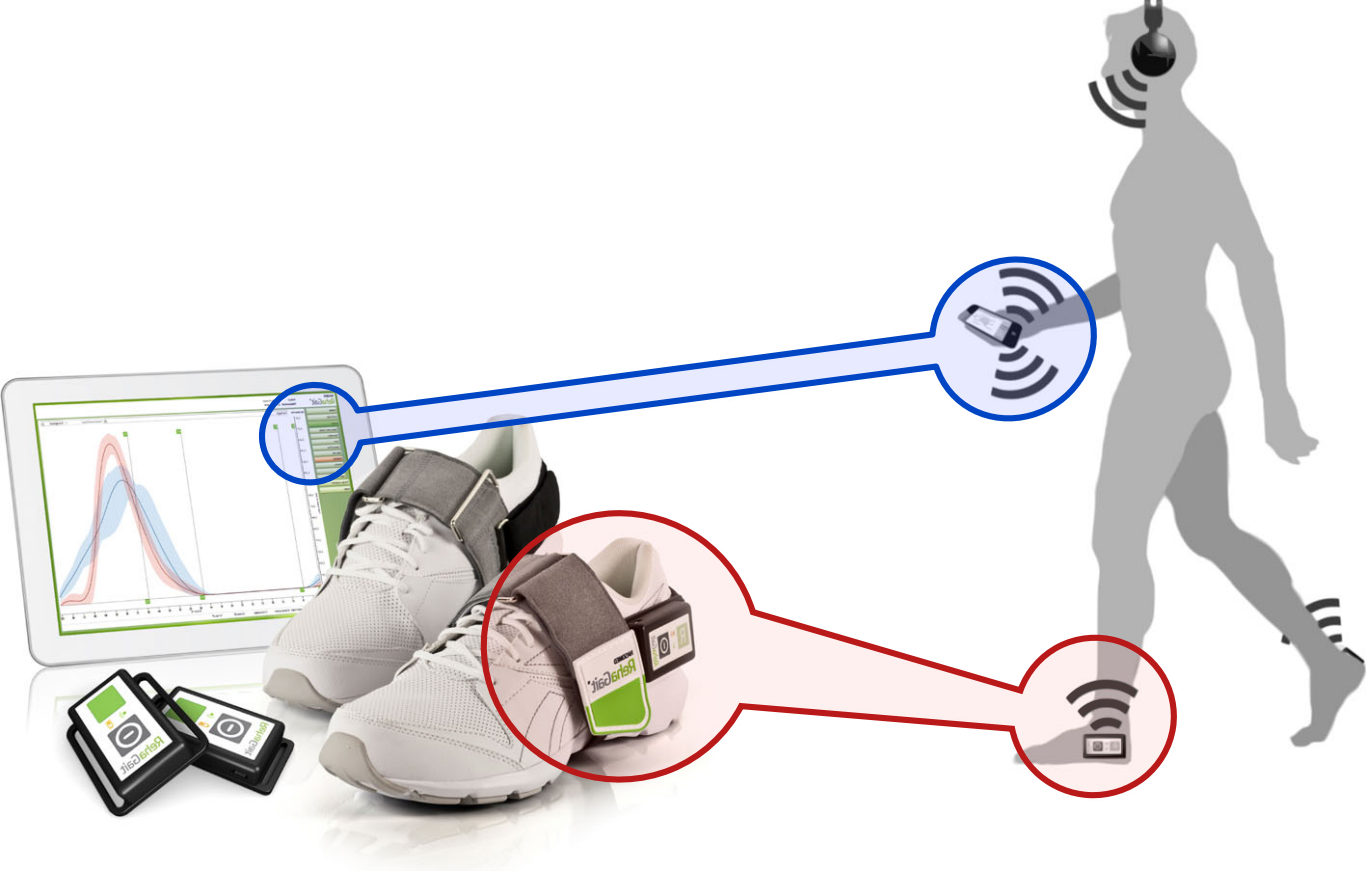
Mobile, inertial measurement unit (IMU)-based gait analysis and feedback system “RehaGait” (Hasomed GmbH, Magdeburg, Germany). Shoe mounted IMUs connect to a tablet computer via Bluetooth, where gait parameters are calculated and compared to their physiological speed-corrected reference in real-time. On the basis of this calculation, automated verbalized feedback is then presented to the user

The IMU-based gait analysis and feedback system RehaGait (HASOMED GmbH, Magdeburg, Germany) was used for this study [38]. It consists of a pair of IMUs mounted to the user’s shoes by straps placing the sensors laterally just below the ankle joint (Fig. 1). The sensors (dimensions: 60 × 15 × 35 mm) contain a 3-axis accelerometer (± 16 g), a gyroscope (± 2000 °/s) and a 3-axis magnetic compass (± 1.3 Gs) [38], which was not used in the study due to large artifacts in indoor applications. The sensors connect to a tablet computer (Samsung GT-P3110, Android version 4.1.2) via Bluetooth, where the stride length, angle between foot and ground at initial contact (referred to as “foot-to-ground angle”), as well as stance and swing time were calculated with a latency of approx. 50 ms. The physiological norm of these spatio-temporal gait parameters is derived from a set of 1860 averaged gait analyses of healthy individuals (age range 5–100 years; 941 females, body height 1.61 ± 0.13 m; 919 males, body height 1.71 ± 0.17 m) [39].

A major challenge for the development of a mobile feedback system was the definition of a universally applicable evaluation scheme for classification and quantification of the deviation from a physiological gait pattern. This evaluation scheme has to fulfill many requirements, among them a feedback-parameter-independent applicability, the ability for initial categorization of individuals independently of the underlying pathology and extent of the gait disorder, the automated adaptation of training difficulty during feedback training and the possibility to evaluate the course of the training.

For this study, a dimension-less, pathology-independent norm-distance measure for quantification of the deviation from a normal gait pattern was implemented. This measure integrates both the difference between the mean of a dedicated feedback parameter from an individual and an age- and body size-adapted norm, as well as the variance of this norm. Based on this norm-distance measure a level structure was defined, following best practice in game design especially in serious games [40, 41]. Only with the introduction of this generally applicable level structure, a pooled data analysis of the three heterogeneous patient groups was possible.

**Fig. 2:**
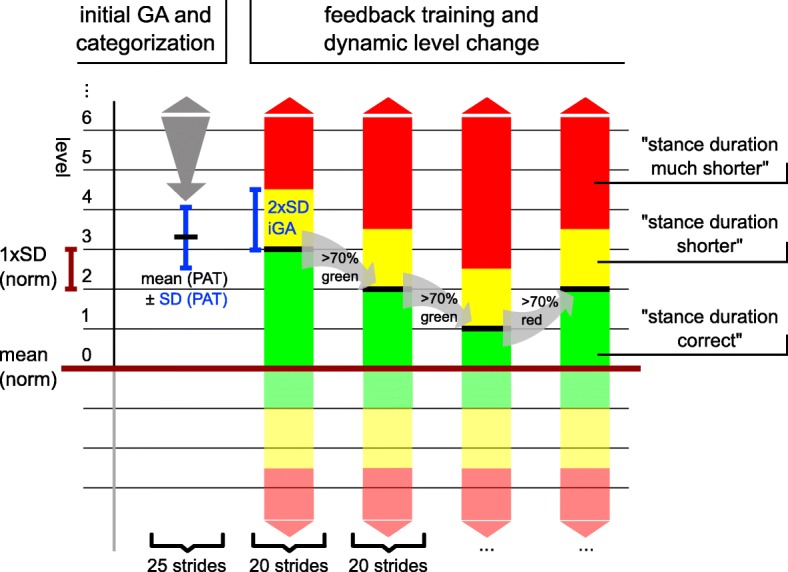
Level structure implemented in the mobile feedback system and exemplary course of levels over training. Mean and standard deviation (SD) of the respective physiological reference (dark red) constitute the absolute training aim and the distance between training levels, respectively. In an initial gait analysis (GA), mean and SD of the user are calculated and used to define the training level at therapy onset together with the level aim and the width of the yellow feedback area (directional feedback). During training, levels change depending on the percentage of feedback values in the red and green range of values, thereby automatically adjusting training difficulty. Training difficulty increases (level decreases) if more than 70% of the feedback values are in the green range over a defined number of strides (20 in this study). In this example, the initial level at training onset was 3 and changed to 2, decreased to 1 and then went up to 2 again. The feedback structure below the physiological mean, graphically represented by the shaded feedback areas, is fixed and follows the structure of level 0. For a better understanding, a situation is depicted in which the mean of the feedback parameter obtained in the initial GA of the patient is higher than the physiological mean. In the opposite situation the feedback structure would be mirrored at the level of the physiological mean

In detail, the range for each level of the norm-distance measure is centered around the mean value of the physiological norm of the respective feedback training parameter and has the size of one standard deviation (SD) of the norm representing the physiological “noise” (Fig. 2). Levels start from 0 with no upper limit. Level 0 represents the lowest level with the least deviation from the norm, while a level n indicates that the current deviation of the mean value ranges between n and n + 1 SDs of the norm. Thus, the level structure forms a positive linear scale for unambiguous assignment of a deviation from the norm to a dedicated level. With this categorization principle, each individual can be assigned a level independent of the gait abnormality.

Feedback about the distance from the normal reference value is categorized into three different ranges: Green (correct), yellow (moderate directional instructions) and red (strong directional instructions) (Fig. 2). The boundaries of these ranges are defined based on the results of the initial gait analysis (iGA), which is performed at the beginning of each training visit. The starting level is set based on the individual’s mean of the feedback parameter obtained during the iGA (Fig. 2). The upper boundary of the “correct”-range - the so called “level aim” - is set by the level boundary below the mean iGA value. The extent of the yellow range equals to twice the SD of the feedback parameter obtained during the iGA. The red feedback range begins at parameter values above the level aim plus twice the iGA-SD and has no upper boundary (Fig. 2). During the training session, after every third stride, a feedback value is calculated by comparing the median of the preceding 3 strides to the current level aim. To feed back this value 3 basic feedback instructions are used according to the above-mentioned categories, e.g., “stance duration much shorter” (red), “stance duration shorter” (yellow) and “stance duration correct” (green) (Fig. 2). Feedback instructions are generated by the tablet computer using the Android-OS default Google text-to-speech engine.

Every 20 strides, the program logic decides on the adaption of the training level: If more than 70% of the feedback values fell into the red area the level is increased, i.e., the difficulty to achieve the level aim is decreased. On the contrary, if > 70% fell into the green area, the level is decreased, i.e., the difficulty to achieve the level aim is increased (Fig. 2). If neither of these two conditions apply, the level remains unchanged for another 20 strides. To prevent overcorrection, the feedback structure past the physiological mean is not changing and always follows the structure of level 0. To prevent guidance effects and encourage self-reflection [42], a fading feedback approach [43] was implemented:

In case a user of the system fulfilled the current movement aim, the frequency of positive feedback was stepwise reduced (feedback output every 3rd, 6th, 15th, 30th, 45th, etc. stride), until directional feedback (red or yellow category) was given. Directional feedback was always provided every 3rd stride.

### Inclusion/exclusion criteria

Legal age, a gait abnormality caused by a stroke or incomplete spinal cord injury (iSCI) or confirmed by an observational gait analysis of a clinical specialist (elderly), the ability to walk for 20 min and at least 100 m in the 6-min walk test [44] were the common inclusion criteria for all groups. In general, all walking aids were allowed, but only ankle-foot-orthoses were allowed as braces. Individuals with iSCI at any neurological level of injury and preserved motor function below the lesion level (American Spinal Injury Association (ASIA) Impairment Scale (AIS) grade C or D [45]) were included not earlier than 6 weeks after onset of paralysis. Individuals with ischemic or hemorrhagic stroke were included not earlier than 3 months after onset of paresis. A Mini Mental-Status-Test [46] was applied in individuals with stroke to screen for cognitive impairments. Individuals with severe dementia (Mini Mental-Status-Test total score < 18) were not included. Individuals with iSCI or stroke were excluded in case of strong spasticity (Modified Ashworth Scale (MAS) [47], unilateral score > = 10) or a history of epileptic seizures. Elderly with an age of at least 65 years and not specifically defined gait abnormalities were included. Subjects with a history of a previous stroke or Parkinson’s disease (freezing and/or shuffling gait hamper gait phase detection with RehaGait) were excluded from the study. Study participants had to perform complete foot clearance during swing phase and a foot-flat phase (both heel and toes are on the ground [48]) during stance, because these are crucial for gait event detection by the RehaGait system. A sample size of 15 individuals per group was intended for feasibility testing. An a-priori sample size calculation was not possible as no effect size of this novel therapy method is known.

Individuals with iSCI and stroke were recruited from the inpatient or outpatient service at the Spinal Cord Injury Center in Heidelberg, Germany. For outpatient recruitment an outpatient call for participation was published. Elderly were recruited in the day care unit of the geriatric hospital of the University of Jena, Germany, during an inpatient stay for general check-up. The recruiting period extended from July 2015 to November 2016. In all individuals, no expense reimbursement was provided for participation. All study participants were instructed to continue their therapy programs over the course of the study.

Prior to study inclusion, written informed consent was obtained from all participants. Ethical approval was granted by the ethics committees of the medical faculty of Heidelberg University (S-168/2015) and Jena University (4377–04/15). The study has been registered with ID DRKS00011853 in the German Register for Clinical Trials (DRKS). The study protocol remained unchanged over the whole runtime of the study.

### Clinical assessments and users’ satisfaction survey

In the iSCI group, neurological impairment was assessed with the International Standards for Neurological Classification of Spinal Cord Injury (ISNCSCI) [45]. The ISNCSCI assessment involves functional testing of 5 key muscles in each of the upper and lower extremities as well as light touch and pin-prick testing in 28 dermatomes, to estimate location and severity of the SCI. Participants with stroke underwent a manual muscle test of key muscles (flexors and extensors of hip, knee and ankle, scores 0–5 as defined in ISNCSCI, referred to as “Mscores”) as well as an ISNCSCI compatible pin-prick testing. For reasons of comparability, the WISCI II [49] was also applied in the stroke group to describe the dependency on walking aids. To test for potential changes in walking ability, the Timed up and go test (TUG) [50] and the 10-m walk test (10MWT) [44] were used. The end user’s satisfaction with the therapy system was obtained post training by a questionnaire based on the Quebec user evaluation of satisfaction with assistive technology (QUEST) [51].

### Study protocol

**Fig. 3:**
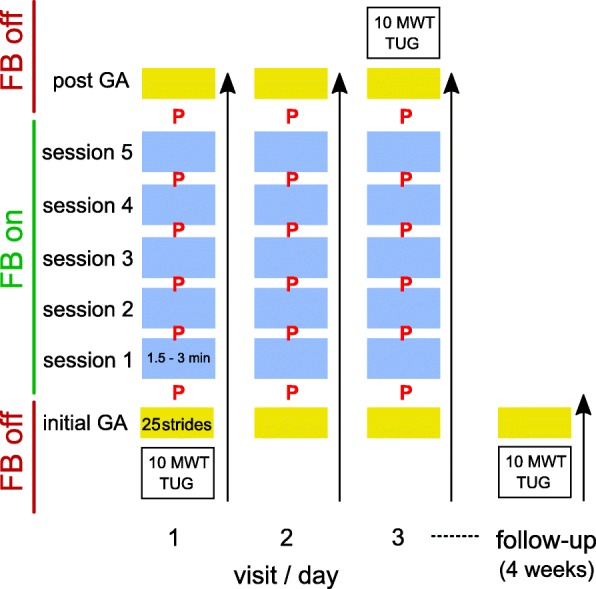
Overview of the study protocol. Gait analyses (yellow, GA) over 25 strides are performed before and after the training, as well as at the follow-up visit. Training sessions (blue) are interrupted by pauses (P) of no more than 2 min each. During gait analyses and walking tests (10-m walk test (10MWT) and Timed up and go test (TUG)) no feedback (FB) is given

The protocol consisted of 3 consecutive training visits and 1 follow-up assessment 4 weeks post training (Fig. 3). Each training visit started with an iGA over 25 strides, followed by 5 sessions of feedback training over 3 min. The first iGA of the first training visit served as the initial baseline for level calculation [52]. For patients who reported not being able to complete a 3-min-session and/or reported mental or physical fatigue, training session duration could be reduced to 2 or 1.5 min based on the therapists’ decision. The minimum total therapy duration for data evaluation was 22.5 min. Each training visit ended with a post gait analysis (pGA). A follow-up assessment was made four weeks after the training phase for the iSCI and stroke groups only. A follow-up assessment of elderly individuals was not possible due to a limited stay (< 3 weeks) in the outpatient clinic of the University of Jena. The 10MWT and TUG were conducted before and after the training and during the follow-up visit. Walking assessments and feedback training were carried out indoors on a barrier-free, even-ground walkway by one assessor in the respective center.

### Training procedure

In each individual, the gait parameter with the highest level, i.e. with the highest deviation from the physiological norm, obtained in the first iGA was chosen as feedback parameter. In case individuals were not able to voluntarily influence the chosen parameter in the resting position, an alternative feedback parameter was chosen. Prior to the first feedback therapy session, individuals were informed about the intent and frequency of the verbalized feedback, and the meaning of a level and its dynamic change.

All walking related assessments were conducted using the same shoes and assistive devices. Assessments were interrupted if individuals reported an increasing fear to fall or if exhaustion jeopardized to safely continue the intervention. For safety reasons, an assistant walked next to the study participants. Participants were free to choose earphones or to listen to the build-in speakers of the tablet computer. Throughout all therapy sessions, study participants were free to choose their comfortable walking speed and the strategy to accomplish the task imposed by the verbalized feedback instructions.

### Data evaluation & statistics

Data from participants who completed the 3 days of training were evaluated. Main outcome parameter was the average level of the trained parameter of iGAs on visits 1 (baseline) through 3 calculated per individual over 25 measured strides, while missing values for single strides were omitted. A Friedman’s test was used to detect overall significance. Bonferroni-corrected Wilcoxon post-hoc tests were used to detect changes between training visits. Since non-parametric tests were used, data is reported by median and 25th and 75th percentiles (median (25, 75%)). For analysis of the overall therapeutic effect additional evaluations were made by calculating the average level per iGA of all levels of parameters not trained (including contralateral body side) in the respective individual.

In order to get an impression about changes in actual values of gait parameters not in the focus of the feedback training (contralateral side and other time-distance parameters), a subgroup consisting of individuals training foot-to-ground angle, the most frequently chosen parameter, was made. These gait parameters (stride length, foot-to-ground angle, stance duration, swing duration) were evaluated over the first 3 iGAs. Walking tests (TUG, 10MWT) were carried out before iGA 1, after pGA 3 and after follow-up (Fig. 3). Gait parameters of the subgroup and walking tests were evaluated using paired Wilcoxon tests.

End user questionnaires were evaluated with descriptive statistics using boxplots and histograms.

Statistical evaluations were performed using R version 3.2.1 [53]. The threshold for significance was α < 0.05. Graphics were generated using the ggplot2 R package [54] and Inkscape version 0.91.

## Results

### Participants

**Fig. 4:**
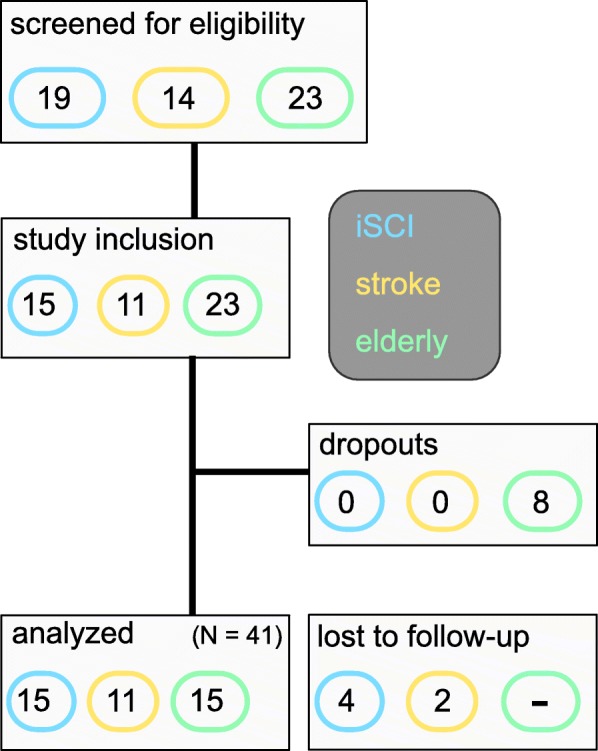
Recruitment process flowchart. Altogether, 41 individuals finished the training period of the study and their data was analyzed. A follow-up was not planned in the elderly group. Colors for patient groups are consistent in all graphs

Fifty-six individuals (19 iSCI, 14 stroke and 23 elderly individuals) were screened for eligibility, 7 of which could not be included due to insufficient walking abilities. Forty-one individuals (15 iSCI, 11 stroke and 15 elderly individuals) participated in all training visits and were included in the analysis. Eight elderly subjects dropped out due to an insufficient number of training sessions (< 5 training sessions/visit) or voluntary withdrawal of consent. Six individuals (4 iSCI, 2 stroke) were lost to follow-up (Fig. 4). Individuals with iSCI (11f, 4 m, 53 ± 18 years, 6 cervical, 6 thoracic, 3 lumbar) were all classified as AIS D at the time point of inclusion (57 ± 132 months post injury) with a lower extremity motor score of 40.7 ± 6.7 (max. 50) (Table 1). Stroke individuals (4f, 7 m, 57 ± 8 years, 8 ischemic, 2 hemorrhagic, one unresolved cause) were included 66 ± 74 months after onset of paralysis. All stroke individuals suffered from hemiparesis (Mscores non-impaired body side: 29.89 ± 0.33 (max. 30), Mscores impaired body side: 21.78 ± 4.35; Table 1). Elderly participants (7f, 8 m) were 81 ± 6 years old. The mean Mini Mental-Status-Test total score was 29.4 ± 0.8 (range 28–30).

**Table 1:** Patient description

group/ID	sex	age	MAI	WISCI II^a^	6-Min-Test	LEMS [50]	PP [112]	AIS	NLI
iSCI1	f	29	24	20	494	46	101	D	L1
iSCI2	f	52	120	13	150.5	31	33	D	C2
iSCI3	f	62	35	20	353	43	81	D	T5
iSCI4	f	49	6	20	331.5	48	95	D	C4
iSCI5	m	61	64	20	445	46	85	D	T6
iSCI6	m	59	4	18	171	41	56	D	C5
iSCI7	f	57	34	13	100	26	68	D	T5
iSCI8	m	72	3	19	359	44	37	D	C2
iSCI9	m	46	3	13	301.5	45	95	D	L1
iSCI10	f	57	1	13	338	40	74	D	T10
iSCI11	f	69	2	9	215	47		D	C4
iSCI12	f	18	13	16	307	41	62	D	T5
iSCI13	f	59	520	18	319.5		90	D	T12
iSCI14	f	77	27	13	299.5	41	42	D	C3
iSCI15	f	20	2	16	245	31	100	D	L2
	mean	52.5	57.2	16.0	295.3	40.7	72.8		
	SD	17.7	132.0	13; 18.5	105.8	6.7	23.7		
						Mscore [30]	PP [56]	hemisphere - artery	
stroke1	f	57	12	20	283	19	23	right	
stroke2	m	53	127	20	500			left - A. cerebri media	
stroke3	m	39	247	15	334.5	24	56	right - A. cerebri media	
stroke4	f	72	9	20	500	27	44	right - A. cerebri media	
stroke5	m	58	65	20	444.5	23	55	left - A. basilis	
stroke6	m	59	23	15	219.5	12	0	right - A. carotis	
stroke7	f	58	33	20	294	23	42	left - A. carotis interna	
stroke8	m	58	130	18	347.5	24	52	right - A. carotis	
stroke9	m	54	39	15	277	20	47	left - A. vertebralis	
stroke10	f	56	31	15	344.5			left - A. cerebri media	
stroke11	m	61	9	20	378	24	54	left - A. cerebri media	
	mean	56.8	65.9	20.0	356.6	21.8	41.4		
	SD	7.7	74.1	15; 20	91.9	4.4	18.6		
elderly1	f	82							
elderly2	m	76							
elderly3	f	73							
elderly4	m	89							
elderly5	f	72							
elderly6	m	77							
elderly7	m	90							
elderly8	f	77							
elderly9	m	82							
elderly10	m	82							
elderly11	m	90							
elderly12	f	84							
elderly13	m	78							
elderly14	f	76							
elderly15	f	86							
	mean	80.9							
	SD	6.0							

Characteristics of individuals with incomplete spinal cord injury (iSCI), after stroke, and with old age (elderly) included in data evaluation. Sex and age are given for all groups. For the group with iSCI, months after injury (MAI), data according to the International Standards for Neurological Classification of Spinal Cord Injury (ISNCSCI), Walking Index for Spinal Cord Injury II (WISCI II), 6-min walk test (6-Min-Test), Lower Extremity Motor Score (LEMS), Pin Prick score (PP), ASIA Impairment Scale (AIS) and Neurological Level of Injury (NLI) are listed. For individuals after stroke, key muscles motor scores (flexors and extensors of hip, knee and foot, motor score grading according to MRC) as well as ISNCSCI pin-prick testing have been determined (Mscore and PP, respectively) and are listed for the affected body side. Maximal scores are displayed in brackets. Additionally, information about the location of the insult (brain hemisphere and artery) is listed for this group

^a^For reasons of consistency, WISCI II scores were given for individuals with stroke, too. For WISCI II scores, median and 1st and 3rd quartile replace mean and SD

### Training parameters and training time

**Fig. 5:**
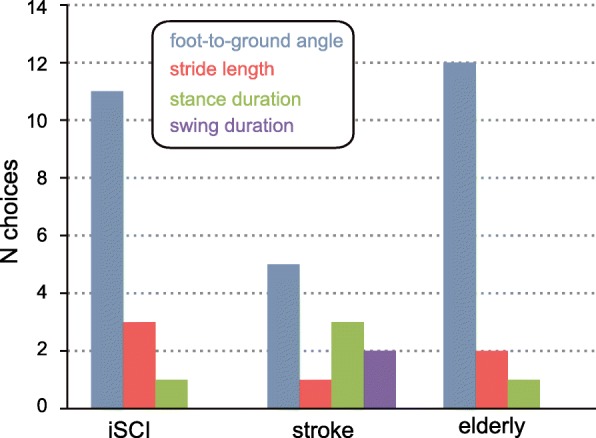
Distribution of training parameters chosen for feedback therapy ordered by group of participants. Foot-to-ground angle is defined as the angle between foot and ground at heel strike. Note that 2 in 11 stroke individuals were trained with feedback of a gait parameter on the unaffected body side (2× stance duration of the unimpaired leg)

Foot-to-ground angle was the most frequently used training parameter in all groups. (Fig. 5). Total average therapy time was 36.3 ± 8.9 min for individuals with iSCI and 37.5 ± 7.4 min for individuals after stroke, respectively. In both patient groups, each participant completed 5 training sessions per training visit. Therapy time for elderly individuals was > 35 ± 4.3 min with a training session time between 2.5 min and 3 min. Four elderly individuals did not complete all 5 training sessions per training visit, but complied with the minimally required total therapy duration.

### Changes in training levels

A pooled analysis of all 3 groups together revealed a significant reduction of the level (= 1 x SD of the reference of the feedback parameter) over time (Friedman’s Test *p* < 0.0001, Table 2). Post-hoc tests between the 3 iGAs of all training visits revealed a significant decrease of the iGA level from visit 1 to visit 2 (median level 5 (3, 6) and 3 (2, 4)), and from visit 1 to visit 3 (median level 2 (1, 4)), respectively.

**Table 2:** Overview of the statistical results

	Friedman’s Test	Wilcoxon post-hoc test		
		iGA 1 < −> 2	iGA 1 < −> 3	iGA 2 < −> 3
overall *N* = 41	*p* < 0.0001 *	*p* < 0.0001 *	*p* < 0.0001 *	*p* = 0.2450
		ci1 = 1.50 ci2 = 2.50	ci1 = 1.50 ci2 = 2.50	ci1 = −3.93e^−5^ ci2 = 1.00
iSCI N = 15	*p* = 0.0004 *	*p* = 0.0027 *	*p* = 0.0047 *	*p* = 0.1883
		ci1 = 1.00 ci2 = 1.00	ci1 = 1.00 ci2 = 2.00	ci1 = −6.45*e^− 5^ ci2 = 2.00
stroke N = 11	*p* = 0.0019 *	*p* = 0.0131 *	*p* = 0.0069 *	*p* = 0.5862
		ci1 = 1.00 ci2 = 3.50	ci1 = 1.00 ci2 = 3.00	ci1 = −1.00 ci2 = 2.00
elderly *N* = 15	*p* = 0.0001 *	*p* = 0.0010 *	*p* = 0.0016 *	*p* = 1
		ci1 = 1.50 ci2 = 3.00	ci1 = 1.50 ci2 = 3.50	ci1 = −2.00 ci2 = 1.50

Overview of the statistical results of the changes of the average training levels over initial gait analyses (iGA) of visits 1 through 3, by participant group. Overall significance testing was achieved with a Friedman’s Test. For post-hoc comparisons, a Bonferroni corrected paired Wilcoxon test was used. Significant results are marked with an asterisk (*)

**Fig. 6:**
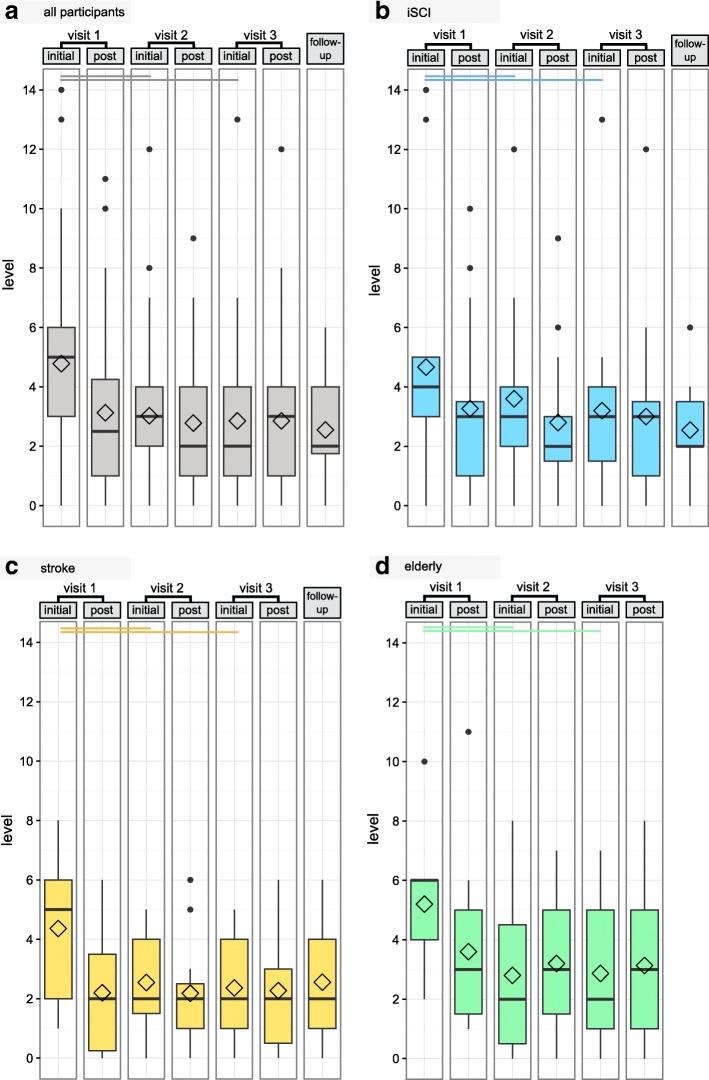
Boxplots with the mean level at initial gait analysis and gait analysis after training (post) on each training visit and at follow-up for all participants (**a**), and separately for the groups with incomplete spinal cord injury (iSCI) (**b**), stroke (**c**) and the elderly (**d**). Diamond shapes indicate mean values. Horizontal bars indicate significant differences according to paired Wilcoxon tests

All groups had a high initial level (iSCI: 4 (3, 5), stroke 5 (2, 6), elderly 6 (4, 6)) and experienced the highest reduction in levels on the first training visit (Fig. 6 and Additional file 1). In the elderly, levels increased during the second and third training day (2 (0.5, 4.5) to 3 (1.5, 5), *p* = 0.1975 and 2 (1, 5) to 3 (1, 5), *p* = 0.4263, respectively, paired Wilcoxon Test) and started with a lower level on visit 2 and 3 compared to the pGA of the previous visit (Fig. 6d, Additional file 1).

From the pGA after training visit 3 to the iGA at follow-up 4 weeks post training, the iSCI group showed a decrease of 1 level, while the stroke group’s level stayed constant.

The average level of all non-trained gait parameters of both body sides did not change significantly over the iGAs, if data of all patient groups were pooled (initial 1.67 (1.17, 3), 1.5 (1, 3) at iGA2, 1.67 (1, 2.67) at iGA3). While individuals with iSCI and the elderly started at moderate values in iGA1 (2 (1.09, 2.83), 2.33 (1.5, 3.83), respectively), stroke individuals showed low initial levels (1.17 (0.92, 1.59)) in all non-trained parameters. A significant reduction (*p* = 0.0096, *N* = 15) of the level was only found in the elderly, in whom levels between iGA1 and iGA2 were reduced from 2.33 (1.5, 3.83) to 1.5 (0.915, 3.33).

### Subgroup gait parameters

**Fig. 7:**
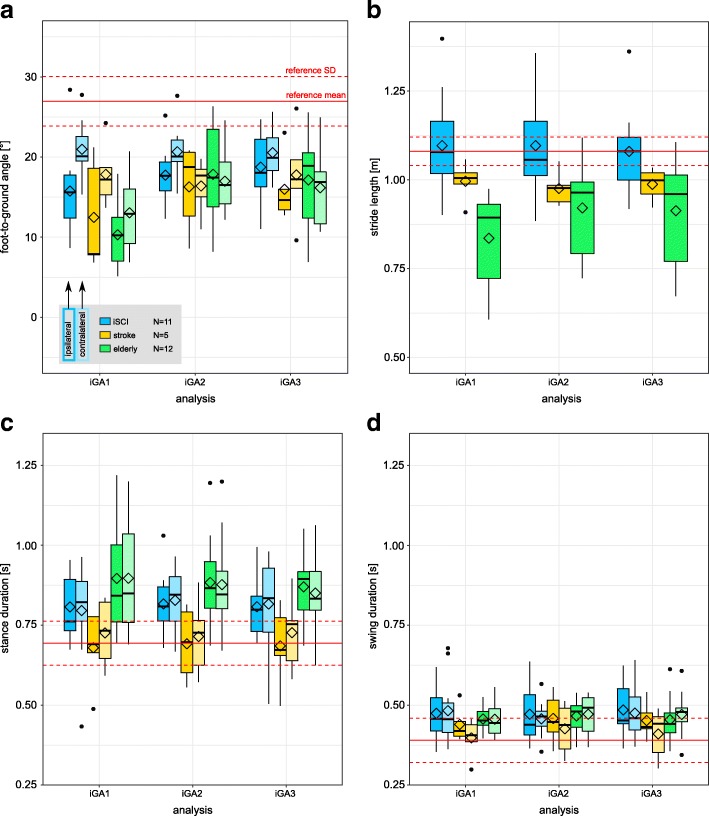
Boxplots showing parameter values “foot-to-ground angle” (**a**), “stride length” (**b**), “stance duration” (**c**) and “swing duration” (**d**) on initial gait analyses (iGA) 1 through 3 of a subgroup of individuals who were trained with “foot-to-ground angle” as training parameter. Ipsilateral and contralateral body sides are plotted separately (dark and light color, respectively). Diamond shapes indicate mean values

Within the subgroup of individuals with iSCI training foot-to-ground angle (iSCI: 11, stroke: 5, elderly: 12), foot-to-ground angles increased significantly towards the physiological reference (*p* = 0.0186, *N* = 11) on the ipsilateral side (Fig. 7) between iGA1 and iGA3, as well as between iGA1 and iGA2 (*p* = 0.0244). On the contralateral body side, no significant increase was found for this group. For the subgroup of individuals with stroke training foot-to-ground angle, no significant changes were found in any gait parameter. The subgroup of elderly training foot-to-ground angle normalized this parameter between iGA1 and iGA3 / iGA2 on the ipsilateral (*p* = 0.001, *p* = 0.0005, respectively) and contralateral (*p* = 0.0005, *p* = 0.0024, respectively) body side. Similarly, stride length increased towards the reference between iGA1 and iGA3 / iGA2 (*p* = 0.0024, *p* = 0.001, respectively).

### Walking test results

The average initial comfortable walking speed captured by the 10MWT before training was 0.91 (0.62, 1.02) m/s in the iSCI group, 1.06 (0.78, 1.19) m/s in the stroke group and 0.71 (0.52, 0.83) m/s in the group of elderly. There were no significant changes in walking speed measured by the 10MWT and time needed for completion of the TUG for either group over the course of the study including follow-up (Additional file 2).

### Questionnaire on user satisfaction

**Fig. 8:**
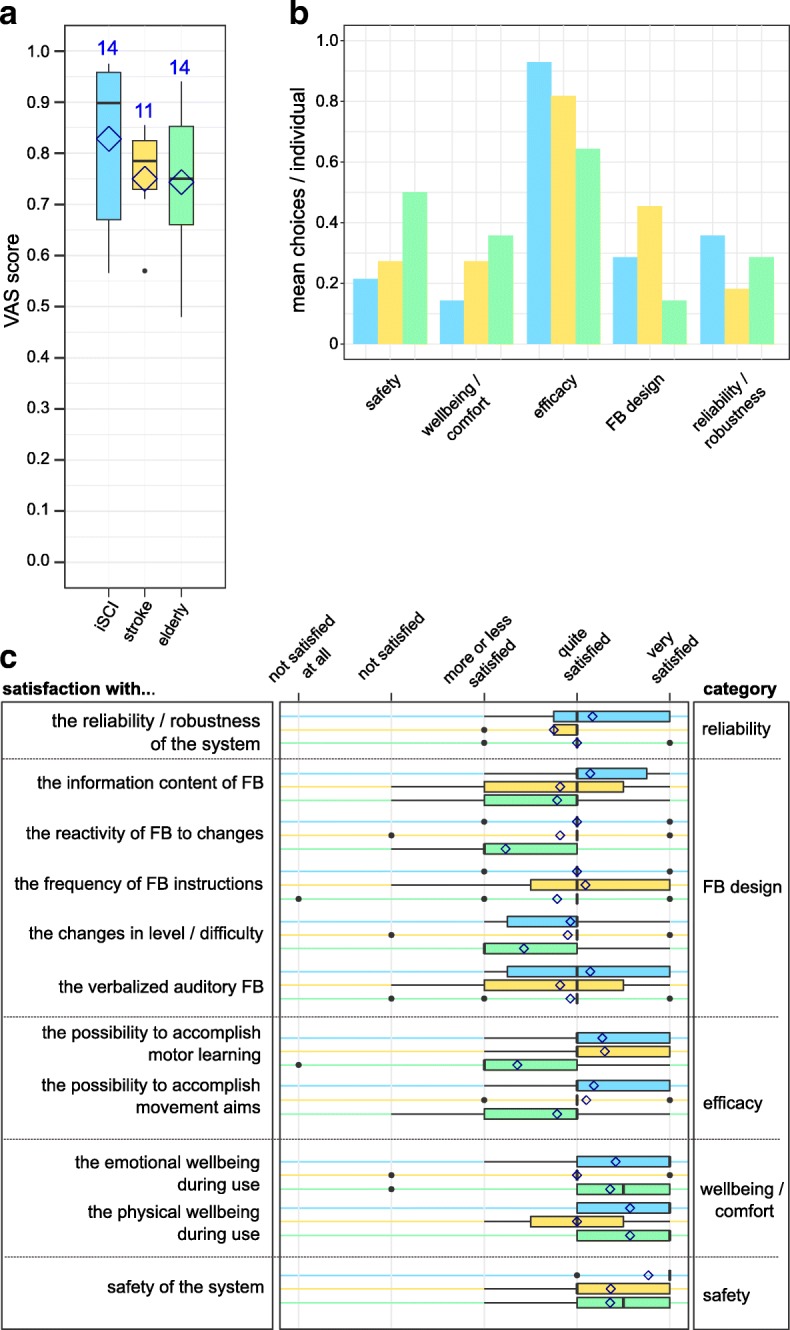
Results of the user’s satisfaction questionnaire (modified Quebec user evaluation of satisfaction with assistive technology (QUEST)). Fourteen individuals with incomplete spinal cord injury (iSCI), 11 with stroke and 14 elderly took part in the survey. Overall satisfaction was captured by a visual analogue scale (**a**), where a score of 1 depicts highest and 0 lowest satisfaction. The categories of the questionnaire rated as most important are displayed in a histogram (**b**). Each participant could choose up to 2 out of 5 categories. Within each category, specific questions could be answered on an ordinal scale from 1 (not satisfied at all) to 5 (very satisfied). These results are displayed using box plots; mean values are displayed using diamond shapes (**c**)

Participants (14 iSCI, 11 stroke and 14 elderly) reported to be overall very satisfied with the system (Fig. 8a), while study participants with iSCI assigned the highest satisfaction scores. Participants were asked to choose 2 out of 5 categories which they rated as most important. Efficacy was chosen most frequently in all 3 groups, while elderly individuals also had a strong focus on safety (Fig. 8b). The responses to category-specific questions yielded overall positive results (Fig. 8c). Elderly individuals were only moderately satisfied with the effect of feedback on the perceived changes on the gait pattern, with the changes in training level, and with the possibility to accomplish long-lasting gait pattern changes. One individual with a stroke associated aphasia suggested the implementation of an optional feedback output using simple sounds instead of polysyllabic words.

## Discussion

The results of our baseline proof-of-concept study demonstrate the feasibility of a mobile, verbalized, real-time gait kinematics feedback therapy using shoe-mounted IMUs. Overall, the introduced level concept was found to be well suited as a universal measure for evaluation of gait abnormalities independent of the underlying pathology as well as for therapy progress monitoring independently of the feedback parameter. The three different groups of study participants comprising gait abnormalities resulting from different etiologies were able to significantly normalize the trained feedback gait parameter over the course of three consecutive therapy visits. In about 2/3 of the study participants, the foot-to-ground angle was used as feedback parameter. A qualitative analysis of the change in levels from iGAs 1 to 3 of the subgroups with feedback parameter “foot-to-ground angle” (*N* = 28) and “time-distance parameters” (pooling all remaining individuals, *N* = 13) suggested that the improvements are not dependent on the trained feedback parameter: In the “foot-to-ground angle” subgroup the median level improved from 4 (3, 6) (median and percentiles 25 and 75) at iGA 1 to 2.5 (2, 3.25) at iGA 2 and finally 2 (1, 4) at iGA3. Similarly, the “time-distance parameters” subgroup started initially at 6(4, 8), reduced the level to 4 (1, 7) at iGA2 and remained at 4 (1, 5) at iGA 3.

Similarly to our previous findings [19] most improvements occurred during the first training visit whereas no significant change was found in the two subsequent training visits. This rapid normalization of the feedback training parameter within minutes indicates that participants were enabled by the automated feedback from RehaGait to make full use of their preserved muscles strength and coordination ability to accomplish the given task. This finding is in line with clinical evaluation results from other mobile feedback systems [55], although a direct comparison of results is not possible due to differences in the technical specifications of the feedback systems and varying study protocols with different outcome parameters [56]. Furthermore, such fast positive effects might be linked to trick movements in proximal joints such as the hip or knee joint. Even though the presence of trick movements cannot be completely excluded, our study participants did not show a deterioration of the non-feedback-trained gait parameters measured on both legs by the shoe-mounted system indicating a true normalization of the overall gait pattern. The results of this proof-of-concept-study underline the fact that a sufficient degree of sensory, either preserved intrinsic or - in case of impaired intrinsic feedback - extrinsic feedback needs to be present for proper motor control [57], an issue which is often not sufficiently taken into account in motor rehabilitation strategies.

The fact that changes of the feedback parameter towards more physiological values occurred very rapidly has some implications not only for the quick identification of non-responders, but also on the future clinical implementation of the automated real-time feedback therapy. It might be more effective to avoid mass practice regimes (e.g., 5 therapy visits in one week) and apply the feedback therapy over an extended period (e.g., 1 therapy visit per week over 5 weeks) of time. Additionally, this helps to prevent a dependency on the external feedback. It might also be possible to consecutively train different gait parameters (potentially associated with different underlying causes of the gait abnormality), while monitoring for increased norm deviation of the parameters not in focus of training. However, these hypotheses need to be confirmed in future studies involving a substantial number of participants.

From a technological viewpoint, the feedback system might support the selection of the feedback parameter in the future by implementation of an automated selection algorithm based on a quantitative analysis of levels of different gait parameters. Additionally, the use of more sensors would allow for an assessment and feedback of the kinematics of multiple joints and thereby controlling for potential trick movements, however, with the drawback of a higher system complexity and longer preparation times.

At follow-up 4 weeks after training, positive carryover effects were still present in individuals with iSCI and stroke, indicating that some motor learning took place. However, it is not known in how far concomitant physiotherapy contributed to this process. On the second and third visit, only in the elderly, the deviation from the norm increased (although not significant) during the instrumented feedback therapy, where study participants have to concentrate on the feedback instructions and simultaneously on the adaption of their gait pattern. This decrease in task performance is known also from other dual-task gait studies [58]. Furthermore, elderly might rely on the recruitment of many brain regions for compensatory purposes during motor tasks [59], thus increasing mental workload and decreasing attention. Therefore, it might be advisable to introduce shorter training sessions in order to avoid mental and/or physical exhaustion specifically in persons with reduced cognitive abilities.

The analysis of the pre-post therapy walking tests revealed no improvements, although all three patient groups had a lower mean initial comfortable walking speed (iSCI: 0.91 m/s, stroke: 1.06 m/s, elderly: 0.71 m/s) compared to non-disabled persons (approx. 1.4 m/s for people in their 50s, approx. 1.3 m/s for people in their 70s) [60]. However, due to the small amount of only 3 training visits, we did not expect a substantial improvement. On the other hand, study participants did not show a slower walking speed, although they were trying to maintain a more physiological walking pattern during the walking tests.

Our evaluation of the RehaGait real-time feedback-system followed a user-centered design approach obtaining feedback from the users about their satisfaction with different aspects of the device. Despite the fact that the walking tests were not able to capture the effects of the feedback therapy on the improvement of the quality of the walking pattern, the overall positive ratings in the users’ satisfaction questionnaire in particular on the therapeutic efficacy of the system can be interpreted as a proof for the perceived benefits of the therapy from the user side. The verbalization of the feedback was well received, however given the substantial number of potential future users with aphasia (20–30% of initial stroke [61, 62]), alternative auditory feedback, e.g., through sounds, should be considered for this group. In general, future studies need to determine the minimal functional requirements of feedback therapy responders. Our study was intended as a proof-of-concept that individuals with different gait disorders including lesions of the CNS and the associated muscular weakness are capable to normalize a selected gait parameter by real-time feedback. If this normalization results in an improved clinical outcome, e.g. reduction of pain or other musculoskeletal complications, needs to be shown in future studies with long-term follow-up. The pre-post-intervention baseline study design was possible for this proof-of-concept study, because the involved individuals with a chronic gait disorder have very limited potential for spontaneous recovery. This would be different in individuals in the acute or subacute stage, where the involvement of a control group is mandatory.

Future work should evaluate in how far the level structure of the norm-distance measure introduced in this work can be used for objective assessment of gait disorders and of effects of interventions. For this, the relationship between the levels and established, clinical, gait disorder etiology-specific, ordinally scaled gait assessment tools such as the Spinal Cord Injury Functional Ambulation Inventory SCI-FAI [63] or the Functional Gait Assessment [64] needs to be determined [65].

### Study limitations

To foster fast recruitment of study participants, the inclusion criteria were very broad resulting in inclusion of all available ambulatory patients without further specifying the pathological gait pattern, which might have resulted in a selection bias.

This analysis of the study results faced a multiple outcome measure and multiple testing problem in an effort to generate a maximum number of hypotheses with the limited number of study participants and assessment visits. Corrections for multiple testing were limited to post-hoc comparisons of the main outcome measure.

## Conclusions

The results of this proof-of-concept study show that a standardized, etiology-independent, mobile feedback therapy based on shoe-mounted IMU sensors is feasible. Providing external real-time feedback about the deviations from a normal gait pattern addresses an important problem in restorative walking rehabilitation programs and may effectively contribute to task-oriented locomotion therapies aiming at the normalization of a non-physiological gait pattern. Future studies are needed to define the frequency and individual implementation of feedback therapy paradigms and should log changes in hip and knee angles.

## Additional files

Additional file 1: Level data for all visits. Level at pre-training (pre) and post-training (post) gait analyses (GAs) on visits 1 through 3 and follow-up arranged by participant group. Median and percentiles 25 and 75 (in braces) are listed. (DOCX 16 kb)

Additional file 2: Results of the 10-m walk test (10MWT) and Timed up and go Test (TUG). Groupwise results of the 10MWT and TUG performed before the 1st gait analysis (GA) on visit 1, after the post GA of visit 3 and at follow-up. Median and percentiles 25 and 75 (in braces) are listed. (DOCX 16 kb)

Additional file 3: Complete dataset of gait parameters and levels measured / calculated by RehaGait. Complete dataset listing average levels per individual per gait analysis, as well as mean gait parameters (stance and swing duration, foot-to-ground angle, stride length) measured by the RehaGait system. (XLSX 58 kb)

Additional file 4: Complete dataset of walk test results. Complete dataset listing results of the 10-m walk test (10MWT) and Timed up and go test (TUG). (XLSX 12 kb)

Additional file 5: Complete dataset of end user survey results. Complete dataset listing results on the questionnaire based on the modified Quebec user evaluation of satisfaction with assistive technology (QUEST). Responses on a 5 point ordinal scale (1 = not satisfied at all, 2 = not satisfied, 3 = more or less satisfied, 4 = quite satisfied, 5 = very satisfied) of category-specific questions are followed by the selection of users’ most important categories (1 = selected). The last column contains the results of a visual analogue scale (VAS, 0–1) rating. (XLSX 13 kb)

## Abbreviations

10MWT: 10-m walk test
AIS: ASIA Impairment Scale
ASIA: American Spinal Injury Association
DRKS: German Register for Clinical Trials
iGA: initial gait analysis
IMU: inertial measurement unit
iSCI: incomplete spinal cord injury
ISNCSCI: International Standards for Neurological Classification of Spinal Cord Injury
MAS: Modified Ashworth scale
pGA: post gait analysis
QUEST: Quebec user evaluation of satisfaction with assistive technology
TUG: Timed up and go test
