# Supplementary material for: Trainer in a pocket - proof-of-concept of mobile, real-time, foot kinematics feedback for gait pattern normalization in individuals after stroke, incomplete spinal cord injury and elderly patients
Source: J Neuroeng Rehabil. 2018 May 29;15:44. doi: 10.1186/s12984-018-0389-4 (PMC5975685; doi:10.1186/s12984-018-0389-4)
Supplement: Supplementary file 1 — Level data for all visits. Level at pre-training (pre) and post-training (post) gait analyses (GAs) on visits 1 through 3 and follow-up arranged by participant group. Median and percentiles 25 and 75 (in braces) are listed. (DOCX 16 kb) [file 12984_2018_389_MOESM1_ESM.docx]

|  |  |  |  |
| --- | --- | --- | --- |
| **visit** | **pre GA**  **level** | **post GA**  **level** | **group** |
| **1** | 5 (3, 6) | 2.5 (1, 4.25) | all |
| **2** | 3 (2, 4) | 2 (1, 4) | all |
| **3** | 2 (1, 4) | 3 (1, 4) | all |
| **follow-up** | 2 (1.75, 4) | - | all |
|  |  |  |  |
| **1** | 4 (3, 5) | 3 (1, 3.5) | SCI |
| **2** | 3 (2, 4) | 2 (1.5, 3) | SCI |
| **3** | 3 (1.5, 4) | 3 (1, 3.5) | SCI |
| **follow-up** | 2 (2, 3.5) | - | SCI |
|  |  |  |  |
| **1** | 5 (2, 6) | 2 (0.25, 3.5) | stroke |
| **2** | 2 (1.5, 4) | 2 (1, 2.5) | stroke |
| **3** | 2 (1, 4) | 2 (0.5, 3) | stroke |
| **follow-up** | 2 (1, 4) | - | stroke |
|  |  |  |  |
| **1** | 6 (4, 6) | 3 (1.5, 5) | elderly |
| **2** | 2 (0.5, 4.5) | 3 (1.5, 5) | elderly |
| **3** | 2 (1, 5) | 3 (1, 5) | elderly |
