# Supplementary material for: Trainer in a pocket - proof-of-concept of mobile, real-time, foot kinematics feedback for gait pattern normalization in individuals after stroke, incomplete spinal cord injury and elderly patients
Source: J Neuroeng Rehabil. 2018 May 29;15:44. doi: 10.1186/s12984-018-0389-4 (PMC5975685; doi:10.1186/s12984-018-0389-4)
Supplement: Supplementary file 2 — Results of the 10-m walk test (10MWT) and Timed up and go Test (TUG). Groupwise results of the 10MWT and TUG performed before the 1st gait analysis (GA) on visit 1, after the post GA of visit 3 and at follow-up. Median and percentiles 25 and 75 (in braces) are listed. (DOCX 16 kb) [file 12984_2018_389_MOESM2_ESM.docx]

| **group** | **pre GA 1** | **post GA 3** | **follow-up** |  |
| --- | --- | --- | --- | --- |
| SCI | 0.91 (0.62, 1.02) | 0.8 (0.66, 0.97) | 0.93 (0.74, 1.01) | 10MWT [m/s] |
| stroke | 1.06 (0.78, 1.19) | 1.05 (0.92, 1.13) | 0.81 (0.74, 1.14) |  |
| elderly | 0.71 (0.52, 0.83) | 0.77 (0.57, 0.83) | NA |  |
|  |  |  |  |  |
| SCI | 13.45 (10.87, 15.59) | 12.79 (10.7, 16.25) | 12.25 (10.23, 15.57) | TUG [s] |
| stroke | 12.48 (10.22, 14.03) | 11.39 (9.57, 14.37) | 10.3 (9.89, 15.23) |  |
| elderly | 17 (12, 22) | 16 (13, 22) | NA |  |
